# JF-305, a pancreatic cancer cell line is highly sensitive to the PARP inhibitor olaparib

**DOI:** 10.3892/ol.2014.2762

**Published:** 2014-12-03

**Authors:** XUELI YANG, CHARLES NDAWULA, HAIYAN ZHOU, XIAOHAI GONG, JIAN JIN

**Affiliations:** 1 School of Pharmaceutical Sciences, Jiangnan University, Wuxi, Jiangsu 214122, P.R. China; 2 National Livestock Resources Research Institute, Tororo, Uganda

**Keywords:** JF-305 cells, olaparib, pancreatic cancer, homologous recombination repair, cell cycle

## Abstract

Poly(ADP-ribose) polymerase-1 (PARP-1) is a DNA nick sensor involved in the base excision repair (BER) pathway. Olaparib, a PARP inhibitor, has demonstrated antitumor activity in homologous recombination (HR)-deficient cancers. To extend this specific therapy to other types of carcinomas, a panel of 11 different cancer cells were screened in the present study. JF-305, a pancreatic cancer cell line of Chinese origin, demonstrated sensitivity to the PARP inhibitor 6(5H)-phenanthridinone. In the present study, 3 μM olaparib conferred a cell survival rate of 25% following four days of treatment. The colony formation efficiency was 83% at 10 nM, and dropped to 12% at 1 μM following seven days of treatment. Furthermore, olaparib induced cell cycle arrest in the S and G_2_/M phases prior to the initiation of apoptosis. Although the incidence of double-strand breaks (DSBs) was increased in the olaparib-treated JF-305 cells, the RAD51 foci were well formed at the sites of γ-H2AX recruitment, indicating an activated HR mechanism. Furthermore, tumor growth was reduced by 49.8% following 22 days of consecutive administration of 10 mg/kg olaparib in the JF-305 xenograft mouse model. In summary, the JF-305 cell line was sensitive to olaparib and provided a prospective model for the preclinical assessment of PARP inhibitors in the therapy of pancreatic cancer.

## Introduction

Poly(ADP-ribose) polymerase-1 (PARP-1) is a DNA nick sensor nuclear enzyme involved in the surveillance and maintenance of genomic integrity. PARP-1 functions in the repair of DNA single-strand breaks (SSBs) via the base excision repair (BER) pathway (1,2). The inactivation of SSB repair by PARP-1 inhibition during the S phase impedes replication fork progression. This leads to replication-associated DNA double-strand breaks (DSBs), which are the most toxic DNA lesions. Therefore, pharmacological inhibitors of PARP-1 may be able to enhance the cytotoxicity of DNA damage agents (3,4). There are currently at least six PARP inhibitors in clinical trial that are being used as chemo/radiotherapy sensitizers (5).

PARP inhibitors were first recognized to be potentially therapeutic by the discovery that PARP inhibition is toxic to cancer cell lines and human tumors with deficient function of homologous recombination (HR), the most important DSB repair pathway. This effect was termed synthetic lethality; when two components act in a co-operating and semi-redundant manner in cell survival, targeting one while the other is defective in a cancer will selectively eliminate the tumor cells, but not be toxic to the normal cells (6). This creates a large therapeutic window.

Olaparib is a well-known PARP inhibitor and has been used clinically in combination therapy for the treatment of multiple cancers (5). Olaparib has advanced into a phase III program as a single treatment for ovarian cancer patients with BRCA gene mutations, which confer HR repair dysfunction in tumor cells (7). Although heterozygous germ-line mutations in the BRCA1/2 genes render a risk of up to 85% for the development of breast cancer, and 10–40% for ovarian cancer, only a small fraction of tumors are BRCA-deficient, accounting for 3–5% of all breast cancers and 15% of ovarian cancers (8,9). This therefore limits the therapeutic utility of olaparib monotherapy. The phase III trial of olaparib has emphasized the requirement for identifying those candidates who are most likely to respond to treatment with the drug (7).

To provide further evidence for clinical application, the sensitivity to olaparib of a range of cancer cell lines, other than the commonly used breast or ovary cell lines, were compared in the present study. Furthermore, the cellular mechanism of the sensitive cell line was preliminarily investigated.

## Materials and methods

### Reagents

6(5H)-phenanthridinone (PHE; Sigma-Aldrich, St. Louis, MO, USA) and olaparib (Selleck, Burlington, USA) were dissolved in dimethylsulfoxide (DMSO) to produce a stock solution of 10 mM, and stored at −20°C for the *in vitro* studies. For the *in vivo* experiment, olaparib was dissolved in phosphate-buffered solution (PBS)/DMSO at 1 mg/ml.

### Cell lines

JF-305 cells were obtained from the Tumor Research Institute of China Medical University (Shenyang, China). MDA-MB-436, Capan-1 and T47D cells were purchased from the Cell Bank of the Chinese Academy of Sciences (Shanghai, China). Rin5f, B16, Acc-3, Patu8988, Bel7402, HNE2, HepG2, DU145, SGC7901 and A549 cells were preserved in the lab. The cells, unless stated otherwise, were maintained in RPMI 1640 medium containing 10% (v/v) fetal bovine serum (FBS). The T47D cells were maintained in the same manner, but supplemented with 0.2 U/ml insulin (Hisun Pharmaceutical Co., Ltd., Taizhou, China). The Capan-1 cells were maintained in Iscove’s modified Dulbecco’s medium containing 20% FBS. The MDA-MB-436 cells were cultured in Leibovitz L-15 medium supplemented with 10% FBS and 0.2 U/ml insulin. The cells were maintained at 37°C in a humidified atmosphere of 5% CO_2_ and 95% air, except for MDA-MB-436, which was cultured at 37°C and in 100% air.

### Clonogenic assay for cell proliferation

Exponentially proliferating cells were plated into six-well plates at a density of 300 cells per well. The following day, the cells were incubated with a series of concentrations of PHE for five days or olaparib for seven days. The cells were fixed and stained with 0.1% crystal violet in methanol/PBS (1:4) and colonies consisting of >10 cells (PHE test) or >50 cells (olaparib test) were subsequently manually counted. The results were calculated as the percentage of colonies in the olaparib treatment group compared with that in the PHE control group.

### CCK-8 assay for cell viability

The cells were seeded into 96-well plates at 1,000–4,000 cells per well depending on the growth rate and left to attach overnight. Olaparib at a concentration of 1 nM-10 μM was added, and the cells were continually incubated for four days (10). The cell viability was measured using Cell Counting Kit-8 (Dojindo, Kumamoto, Japan).

### Foci formation of γ-H2AX and RAD51 by co-immunostaining

The cells were seeded onto sterile confocal dishes and exposed to a medium containing 3 μM olaparib, or PBS, for 24 h. The cells were fixed in pre-chilled methanol/acetone (7:3) at −20°C for 10 min. Subsequent to air-drying, the dishes were washed three times with PBS and blocked using 5% skimmed dry milk and 0.1% Triton X-100 in PBS at room temperature for 1 h. Samples were then incubated overnight at 4°C with mouse anti-phospho-Histone H2AX (Ser139) monoclonal antibody (Millipore, Billerica, MA, USA: dilution, 1:50) and rabbit anti-RAD51 polyclonal antibody (Santa Cruz, Dallas, TX, USA: dilution, 1:50) (11). Subsequent to being washed, the cells were incubated with secondary Cy3-labeled goat anti-mouse immunoglobulin G (IgG), and Alexa Fluor 488-labeled goat anti-rabbit IgG (H+L) antibodies (Beyotime, Suzhou, China), for 1 h at room temperature and protected from light. Subsequent to being washed again, the nuclei were stained with 1 μg/ml DAPI (Beyotime) for 10 min. Images were obtained with a confocal laser scanning microscope (Leica TCS SP8; Leica, Wetzlar, Germany).

### Cell cycle analysis

The cells were plated in six-well plates at concentrations determined to reach 70–80% confluence when analyzed. Following attachment, the cells were incubated with 0, 0.3 or 3 μM olaparib for 48 h, then washed twice with PBS, treated with trypsin and centrifuged at 800 × g for 5 min. The cells were fixed with 70% ethanol for 2 h at 4°C and the pellet was then removed from the ethanol and washed twice in ice-cold PBS. The cell pellet was resuspended in PBS with 50 μg/ml RNase at 37°C for 30 min, followed by 50 μg/ml propidium iodide in the dark at 4°C for 30 min. Samples were analyzed using a flow cytometer (BD FACSCalibur; BD Biosciences, San Diego, CA, USA) and data was analyzed with ModFit software (Verity Software House, Inc., Topsham, ME, USA).

### Nucleus staining and photomicrography

The cells were exposed to olaparib for four days, washed and fixed, and then stained with 1 μg/ml DAPI for 10 min. Images were captured by a video camera (Nikon Coolpix 54, Nikon, Tokyo, Japan) mounted on a Leica CME microscope.

### Xenograft tumor studies

CByJ-Cg-Foxn1nu/Nju mice (male, aged 3–4 weeks) from Nanjing Biochemical Research Institute of Nanjing University (Nanjing, China) were used in the xenograft experiments. The protocol was approved by the Animal Ethics Committee of Jiangnan University (Wuxi, Jiangsu, China). The mice were maintained and handled in isolators under specific pathogen-free conditions and were inoculated to the right axillary cavity with 5×10^6^ cells in 0.1 ml of medium without serum. Tumor volumes were estimated using the formula: Tumor volume = (length / 2) × (width^2^) (10). When the mean tumor volume reached 150 mm^3^, the tumor-bearing mice were randomly split into two groups, with six animals in each group. Mice in the test group received 10 mg/kg olaparib once daily for 22 consecutive days, whilst those in the vehicle group received PBS as a vehicle containing the same concentration of DMSO. The tumor volumes were measured every three days and the established tumors in each animal were individually normalized to their size at the start of the treatment administration. The relative tumor volume (RTV) was calculated according to the formula (12): RTV = TVx / TV0, where TVx is the tumor volume on any given day and TV0 is the tumor volume at the initiation of dosing (i.e., day 0).

### Statistical analysis

Results are presented as the mean ± standard deviation. All statistical analyses were performed using GraphPad Prism version 5.0 for Windows Software (GraphPad Software, La Jolla, CA, USA). Statistical differences were determined by two-tailed Student’s t-test unless stated otherwise. P<0.05 denotes a statistically significant difference.

## Results

### JF-305 cells are hypersensitive to PARP inhibitors

**Figure 1:**
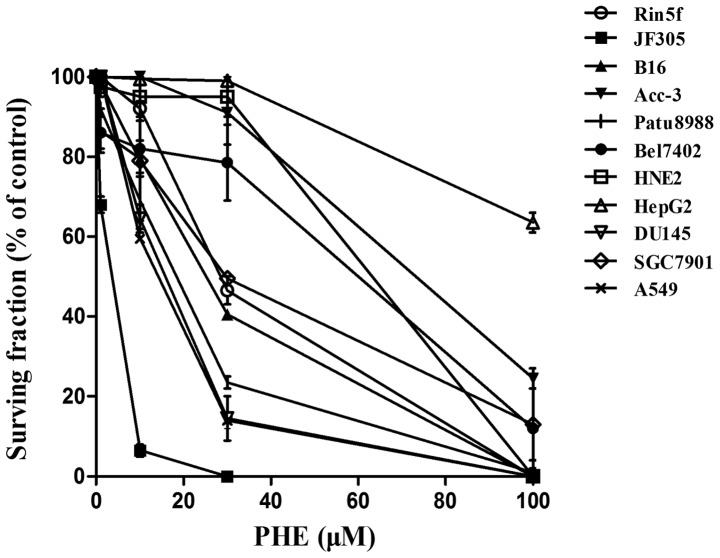
Compared with other cell types, JF-305 cells demonstrate relatively high sensitivity of their colony formation efficiency to the PARP inhibitor PHE. Rin5f, islet tumor cell; B16, skin melanoma cell; Acc-3, salivary gland adenoid cystic carcinoma cell; Patu8988, pancreatic cancer cell; Bel7402, hematoma cell; HNE2, nasopharyngeal carcinoma cell; HepG2, hepatocellular carcinoma cell; DU145, prostate cancer cell; SGC7901, gastric cancer cell; A549, lung adenocarcinoma cell; PHE, 6(5H)-phenanthridinone. Data are expressed as the mean ± standard deviation (n=3).

In the present study, the effects of the PARP inhibitor, PHE (IC_50_, 350 nM) (13), on the colony formation efficiency of various cell lines was investigated. The pancreatic cancer JF-305 cell line was identified to be the most sensitive to PHE, as its colony formation efficiency decreased to <10% when treated with 10 μM PHE, a concentration at which other cell lines retained at least 60% colony formation efficiency (P<0.05) (Fig. 1).

**Figure 2:**
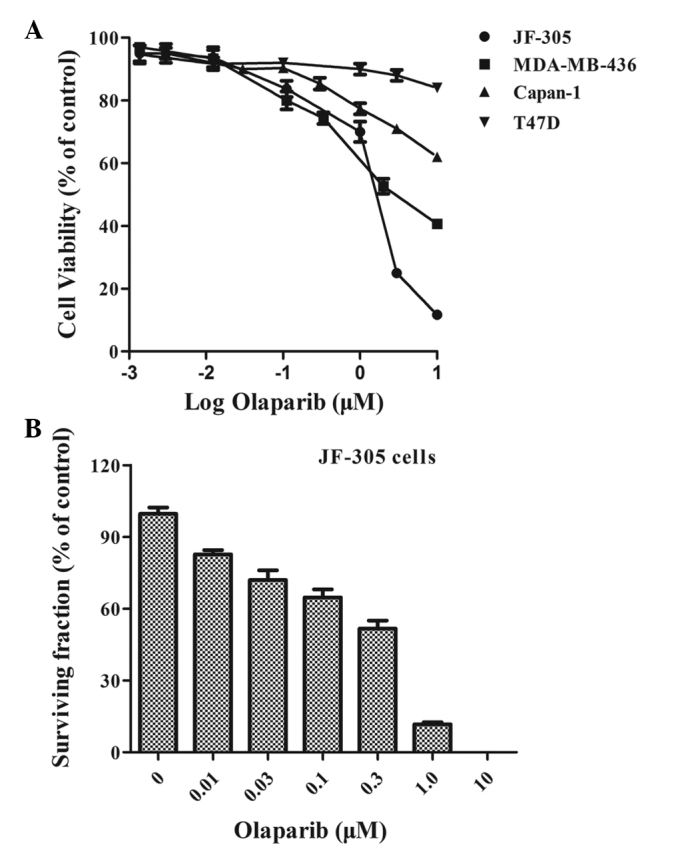
JF-305 cells are sensitive to olaparib. (A) Olaparib selectively inhibited the growth of the JF-305, MDA-MB-436 (BRCA-1 deficient) and Capan-1 (BRCA-2 deficient) cells, but not the T47D (BRCA-1 and BRCA-2 proficient) cells. (B) The colony formation efficiency of the JF-305 cells was inhibited by increasing concentrations of olaparib. Data are expressed as the mean ± standard deviation (n=3).

A more potent PARP inhibitor, olaparib (IC_50_, 5 nM) (10), was then used to confirm this result. For the *in vitro* cell viability assay, the T47D cells (BRCA-1- and BRCA-2-proficient), MDA-MB-436 cells [BRCA1 (5382insC) mutated] and Capan-1 cells [BRCA1 (6174delT) mutated] (10,13) were used as controls. The JF-305 cells exhibited hypersensitivity to olaparib, with a percentage viability of ~25% at 3 μM and 11% at 10 μM, compared with 50% and 41%, respectively, in the MDA-MB-436 cells, and 71% and 62%, respectively, in the Capan-1 cells. The T47D cells, however, demonstrated very little response (P<0.05) (Fig. 2A). To validate the sensitivity of the JF-305 cells to olaparib, a clonogenic assay was performed as a ‘gold standard’ to assess cell proliferation. The colony formation efficiency of the JF-305 cells was significantly reduced upon treatment with an increasing concentration of olaparib (P<0.05). The dosage at which 50% of the cells survived was 0.4 μM (Fig. 2B).

As the aforementioned data demonstrated (Figs. 1 and 2), the JF-305 cells were sensitive to PARP inhibitors *in vitro*.

### Olaparib results in DSBs and cell cycle arrest with activated HR repair in JF-305 cells

**Figure 3:**
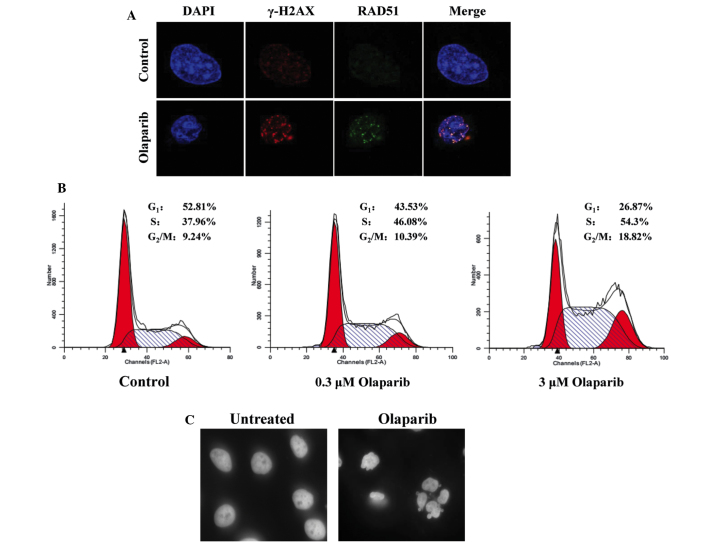
Olaparib induces the DNA damage, cell cycle arrest and cell death of JF-305 cells. (A) Co-immunostaining reveals DSBs and HRR represented by γ-H2AX and RAD51 foci formation in the JF-305 cells exposed to olaparib or a vehicle. (B) S and G_2_/M cell cycle phases were arrested by increasing concentrations of olaparib and analyzed by flow cytometry. (C) DAPI-stained nuclei analysis of JF-305 cells incubated with olaparib and a vehicle. DSBs, double-strand breaks; HRR, homologous recombination repair.

Olaparib targets PARP-1, a component of the BER pathway. Blockage of the BER pathway will induce a large number of potentially lethal DSBs when encountered by replication forks. The nuclear γ-H2AX foci occur at the sites of DSBs (14), therefore, the present study identified γ-H2AX foci in the JF-305 cells to reveal the existence of DNA damage. Treatment of JF-305 cells with olaparib increased the formation of γ-H2AX foci in the nucleus compared with the control (Fig. 3A), which indicated the interaction of olaparib with a functional DNA sensor. In addition, the formation of RAD51 foci, which play a key role in DNA HR during DSB repair, were investigated in the present study. The co-immunostaining analysis revealed that the increased RAD51 foci overlapped at the sites of DSBs (Fig. 3A), which identified activated HR repair in JF-305 cells treated with olaparib.

To determine how olaparib leads to the decrease of cell viability and colony formation efficiency, the cell cycle of the JF-305 cells was analyzed in the present study. Following 48 h of exposure, 3 μM olaparib elicited a 2-fold accumulation of tetraploid DNA content in the JF-305 cells, indicating an arrest in the G_2_/M phase of the cell cycle. The number of cells in the S phase also increased by 43% compared with the untreated group (Fig. 3B). Following 96 h of treatment with olaparib, the DAPI-stained JF-305 cells demonstrated an increase in apoptosis, which usually manifests with chromatin condensation and nuclear fragmentation (15) (Fig. 3C).

Together, this data suggested that despite activated HR, olaparib induced DNA DSBs by PARP inhibition, initiated S and G_2_/M cell cycle arrest and ultimately induced the cells to undergo apoptosis.

### JF-305 tumor growth is delayed by olaparib in vivo

**Figure 4:**
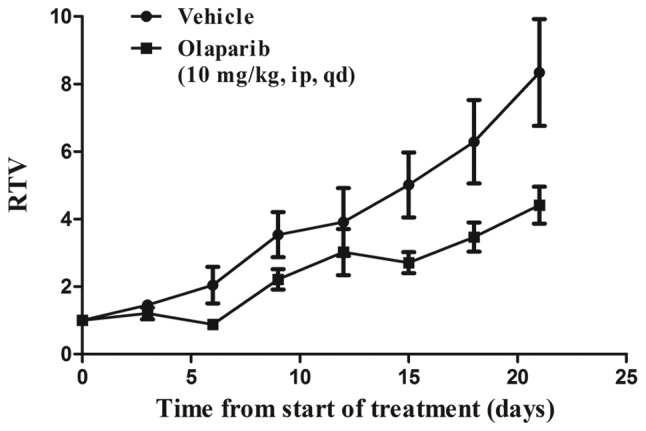
Olaparib delayed the growth of the JF-305 tumors *in vivo* compared with the vehicle. RTV, relative tumor volume; ip, intraperitoneal injection; qd, once daily. Results are presented as the mean ± standard deviation (n=6).

The results from the present study demonstrated that the JF-305 cells were sensitive to olaparib *in vitro*. In addition, JF-305 cells have also previously been reported to exhibit tumorigenicity when transplanted into nude mice (16). In the present study, upon assessment of the response of JF-305 tumors to olaparib *in vivo*, tumor formation was detected after two weeks of inoculation. The mean tumor volume in the control group increased to 1,368 mm^3^ by the 5th week, and to 687 mm^3^ in the olaparib-treated group (P<0.05), a 49.8% reduction following 22 consecutive days of administration (Fig. 4).

The comparable activity of olaparib upon the JF-305 cells *in vitro* and the JF-305 tumors *in vivo* supports a therapeutic model for the preclinical study of pancreatic cancer and the potential applications of olaparib in an Asian population.

## Discussion

Given the potential of olaparib as a therapeutic approach for the treatment of cancer, certain studies have demonstrated interest in the concept of synthetic lethality in cancers with defects in DNA metabolic processes other than HR repair. Previous studies have confirmed that a loss of function of RAD51C, XRCC3, PTEN, ATM, CHK1 or CHK2 (17–20) causes cells and tumors to become sensitive to PARP inhibition. High-throughput RNA interference analysis also identified DDB1, XAB2 and CDK12 as novel genetic determinants of PARP inhibitor sensitivity (21,22). Furthermore, cells deficient in the aforementioned genes became an ideal model for the preclinical study of cancers. The present study identified a cell line, JF-305, which was hypersensitive to olaparib. Although olaparib induced an increase in DNA DSBs, HR was effectively activated. Furthermore, the PARP inhibitors, KU0058684 and olaparib, have previously been shown to arrest the cells in phase G_2_ of the cell cycle in wild-type cells, an effect that was enhanced in BRCA1/2- or RAD51C-deficient cells (23,24). In addition to G_2_/M phase arrest, JF-305 cells also exhibit arrest at the S phase following treatment with olaparib. These factors may indicate a novel molecular mechanism contributing to the sensitivity of PARP inhibitors in JF-305 cells. Identifying the cellular events that occur following the accumulation of RAD51 foci, prior to the cell progressing through the cell cycle checkpoints, will be the objective of a future study.

Pancreatic adenocarcinoma is currently the fourth most common cause of cancer-related lethality. The disease incidence is almost equal to the disease mortality due to a high resistance to chemo/radiotherapy and a poor prognosis (25). The standard first-line therapy for pancreatic adenocarcinoma of gemcitabine alone, or in combination with fluorouracil, demonstrates limited efficacy (26). Attempts have been made to improve the outcome for BRCA-mutated pancreatic cancer by using the PARP inhibitor veliparib, or combining gemcitabine with olaparib or veliparib (6). The results from the present study revealed that the pancreatic JF-305 cell line was sensitive to olaparib as a single treatment either *in vitro* or *in vivo*. This finding further confirmed the clinical potential of olaparib as a combined treatment or monotherapy for pancreatic cancers without a BRCA/HR deficiency.

Furthermore, as is the case for BRCA mutations, the biomarkers of PARP inhibitor sensitivity differ between Asian and Western populations (27,28). It is also unclear how representative the Western findings on pancreatic cancer are for Asian populations (29). The JF-305 cells of Chinese origin from the present study may support an accurate model for PARP inhibitor sensitivity and pancreatic carcinoma in an Asian population.

In conclusion, the present study identified that JF-305, a pancreatic cancer cell line of Chinese origin, was sensitive to olaparib *in vitro* and *in vivo*. Although HR repair was effectively activated in the cells treated with olaparib, the cell cycle was arrested in the S and G_2_/M phases following numerous DSBs. In addition to the regional diversity of gene mutations, this may indicate another functional impairment mutation in JF-305 cells that has the potential to be a model for the preclinical investigation of pancreatic cancer chemotherapy with PARPs as a target.
